# Supplementary material for: Chromothripsis during telomere crisis is independent of NHEJ, and consistent with a replicative origin
Source: Genome Res. 2019 May;29(5):737–49. doi: 10.1101/gr.240705.118 (PMC6499312; doi:10.1101/gr.240705.118)
Supplement: Supplemental Material [file supp_gr.240705.118_Supplemental_file_1.zip › contigs/annotated_contigs/DB116/contig.3.DB116_length_575_mean_cov_8.82782608696.docx]

**DB116_length_575_mean_cov_8.82782608696**

```
TACTGGATATATACCCAAAGGACTATAAATCATGCTGCTATAAAGACACATGCACACATATGTTTATTGCGGCACTATTCACAATAGCA
 >chr9:28481902-28482140 - E=2e-128
AAGACTTGGAACCAATCCAAATGTCTAACAATGATAGACTGGATTAAGAAAATGTGACACATATACACCATGGAATACTATGCAGCCAT

AAAAAATGATGAGTTCATGTCTTCTGTAGGGACATGGATGAAATTGGAAATCATCATTC|T|TGACCTACAATGATTATAATAGCAACT
                                                            >chr9:28479941-28480115 - E
CTTTGTAAACGTAAATGAACCTCACAGAAATCATAGCATTACATAATCTATCAAAACAAATAATAGCAATTTATTATGGTGGTTGAAAT
=5e-90
TTTGCTTAGGAAGTAACCCTATTTAAGTTTCTCTCAAAATGTACATATATATATATA|CACACAT|ACACACACACACACACACACACA
                                                                 >chr9:28479721-2847987
CACACACACACAGATGACATGGTTCCAGTAGTTAAGACATTCTTCAGTATGGTACTTATATGGGAGAAATAGTCATTATAGATAGGACC
8 - E=9e-83
AATGACTGTTCTTTAAAGGAACTAGACACATGTTCCTGAAATACA
```
